# A Simple Equation to Estimate Maximal Oxygen Uptake in Older Adults Using the 6 min Walk Test, Sex, Age and Body Mass Index

**DOI:** 10.3390/jcm12134476

**Published:** 2023-07-04

**Authors:** Peter Šagát, Zvonimir Kalčik, Peter Bartik, Ľuboslav Šiška, Lovro Štefan

**Affiliations:** 1 Sport Sciences and Diagnostics Research Group, GSD/Health and Physical Education Department, Prince Sultan University, Riyadh 11586, Saudi Arabia; sagat@seznam.cz (P.Š.); pbartik@psu.edu.sa (P.B.); 2 The Home of War Veterans, 10000 Zagreb, Croatia; zvonimirkalcik@gmail.com; 3 Faculty of Education, Catholic University in Ružomberok, 034 01 Ružomberok, Slovakia; luboslav.siska@ku.sk; 4 Department of General and Applied Kinesiology, Faculty of Kinesiology, University of Zagreb, Horvaćanski zavoj 15, 10000 Zagreb, Croatia; 5 Department of Physical Activities and Health Sciences, Faculty of Sports Studies, Masaryk University, 625 00 Brno, Czech Republic

**Keywords:** aerobic capacity, functional endurance, old age, walk test, regression equation

## Abstract

Purpose: The 6 min walk test (6MWT) is used in clinical and epidemiological practice as a simple tool to evaluate the maximal aerobic exercise capacity (VO_2_max). To date, little evidence has been provided regarding regression equation models to predict VO_2_max in older adults. Therefore, the main purpose of the study was to develop a reference equation to estimate objectively measured VO_2_max, based on the 6MWT, sex, age and body mass index (BMI). Patients and Methods: In this observational prospective study, we collected the data from 233 asymptomatic participants aged 60–80 years (52.4% women). VO_2_max and the 6MWT were measured using standardized protocols. BMI was calculated as weight (kg) divided by height squared (m^2^). To be able to develop the predictive equation for VO_2_max, we used linear generalized estimating equations (GEE). Results: Older men performed better in VO_2_max (30.9 ± 8.8 vs. 26.3 ± 6.3 mL/kg/min, *p* < 0.001) and the 6MWT (673.9 ± 58.7 vs. 642.3 ± 48.0 m, *p* < 0.001), compared to women. VO_2_max was correlated with the 6MWT (*r* = 0.71, *p* < 0.001), sex (*r* = −0.29, *p* < 0.001), age (*r* = −0.62, *p* < 0.001) and BMI (*r* = −0.38, *p* < 0.001). The model to predict VO_2_max included: VO_2_max (mL/kg/min) = 59.44 − 3.83 *, sex (1—men; 2—women) − 0.56 *, age (years) − 0.48 *, BMI (kg/m^2^) + 0.04 *, and the 6MWT (m) (*R* = 0.85; *R*^2^ = 72.3%, *SEE* = 3.99 mL/kg/min, *p* < 0.001). Conclusion: The newly developed regression equation can be a guideline in clinical and epidemiological practice to predict the VO_2_max in apparently healthy older adults.

## 1. Introduction

Objective methods to assess aerobic capacity represent a key factor for the initial screening and monitoring of changes in functional independence at old age [1]. The use of cardiorespiratory fitness (CRF) for intervention purposes has been considered as a vital sign of cardiovascular health [2]. The ‘golden standard’ of cardiopulmonary exercise testing (CPET) for measuring aerobic exercise capacity is maximal oxygen uptake (VO_2_max) [3,4]. VO_2_max is considered a reliable and valid marker which can predict cardiovascular and all-cause mortality [5,6]. However, the direct measure of VO_2_max is relatively expensive and time-consuming [7], and in low-resource settings, a field-based alternative, like the 6 min walk test (6MWT), is often applied [2,4,7,8].

The 6MWT Is a cost-effective, simple and easy-to-administer tool for assessing the functional endurance capacity [8], primarily in patients with moderate and severe heart and lung diseases [9,10,11]. Due to its applicability in both symptomatic and asymptomatic populations [12], a substantial body of evidence has tried to examine the predictive ability of the 6MWT against the VO_2_max [7,9,10,11,13,14,15,16,17,18,19,20,21,22,23,24,25,26]. Most research has been conducted in diverse groups of patients with various cardiac, circulatory and pulmonary disorders [9,10,11,13,14,15,16,17,18,19,20,21], while only a few studies included healthy individuals [22,23,24,25,26]. In general, a systematic review by Ross et al. [7] has shown a strong correlation between the 6MWT and VO_2_max (*r* = 0.59), with a standard error of estimate (SEE) of 3.82 mL/kg/min. However, such an equation was derived from participants with diseases, and cannot be fully used in healthy individuals. Studies conducted in asymptomatic adults have used multiple regression approach and, in addition to the 6MWT, prediction models included sex [24], age [22,23,24], weight [23,24], height [23,24], body mass index (BMI) [23] and resting heart rate (HR) [23,24]. Although the aforementioned predictors explained between 70% and 80% of the VO_2_max, the development of regression equations for apparently healthy older adults to predict the VO_2_max has been studied less. The available literature on the same topic has used a relatively small sample size with a great age range [22,23,24], which may not be generalizable to other populations. Given the importance of the 6MWT in clinical and epidemiological settings, it is necessary to establish a predictive model for VO_2_max from simple demographic and anthropometric parameters.

Therefore, our purposes were two-fold: (i) to examine the correlation between the 6MWT with VO_2_max; and (ii) to create prediction equations for the 6MWT and VO_2_max in combination with easily administrated demographic (sex and age) and anthropometric (BMI) variables. We hypothesized that the 6MWT would be strongly correlated with the VO_2_max and that adding of further variables such as sex, age and BMI, would improve its predictive ability.

## 2. Material and Methods

### 2.1. Study Participants

**Figure 1:**
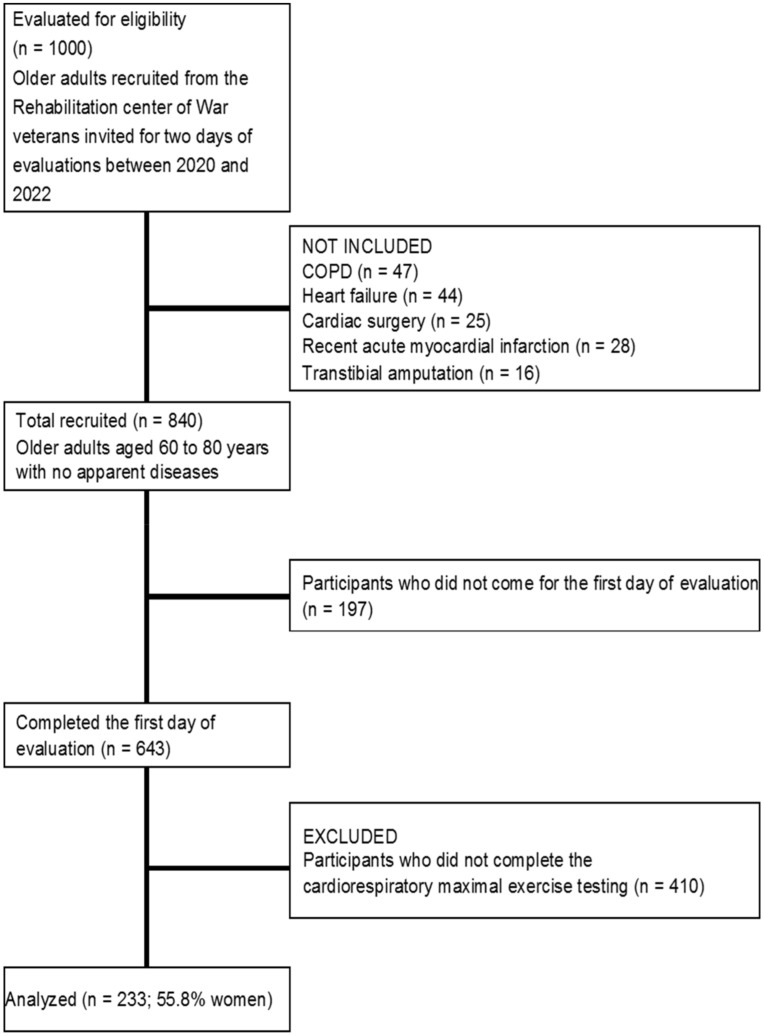
The flowchart diagram of the study.

In this cross-sectional study, the eligible sample size was 1000 men and women aged between 60 and 80 years. The inclusion criteria for participation in the study included self-reported information on: (i) being without chronic diseases, which included chronic heart disease, rheumatic arthritis, chronic kidney disease, stroke, cancer and chronic obstructive pulmonary disease; (ii) the absence of a serious physical or mental illness; and (iii) having all the study variables tested. More detailed information about the recruitment at different stages is presented in Figure 1. Of note, we performed the sample size calculation to estimate the achievable number of participants being adequate for further analyses. With an a priori *t*-test to evaluate the required sample size for the linear regression model, a statistical power of 95%, four predictors, an effect size of 0.15 and *p* < 0.01, the appropriate sample size was 182. Before data collection started, all participants were informed about the aim, hypotheses and methodology of the study. The participants were ensured confidentiality, informed that their participation was voluntary, and that they had the right to withdraw at any time. All participants read and signed the informed consent forms. We followed the methods of the principles of the Declaration of Helsinki [27] and the Ethical Committee of The Home of War Veterans approved the study (Ethical code number: 2022/4).

### 2.2. VO_2_max Assessment

Cardiopulmonary exercise testing (CPET) was performed on a treadmill, using a 10-stage ramp protocol to determine VO_2_max. For the purpose of this study, the testing protocol was programed for each individual to achieve fatigue-limited exercise, with a duration between 8 and 12 min [28]. Following the modified Bruce protocol [29], each participant was approached with individualized increases in speed (max. 1.5 m/s) and inclination (max. 15%) every 2 min until exhaustion, determined by respiration or physical appearance [30]. Of note, these recommendations have been proposed in patients with cardiovascular acute and chronic diseases, so the applicability may be limited to this population. The test was conducted in the same conditions, with a room temperature of 23 °C. The Cosmed K4b^2^ (COSMED, Rome, Italy) was used to analyze and calculate VO_2_max as the highest VO_2_max measured over a period of 15 s near the cessation of the test, and before the recovery phase [24]. All tests were supervised by a certified exercise physiologist. All participants were instructed not to ingest caffeine for 12 h or eat within 3 h prior to CPET, and to come to the test in clothes suitable for performing the exercise.

### 2.3. The 6MWT

The test was conducted using a 30 m straight corridor with a flat, firm ground, and with two cones placed at each end of the course. We followed the testing procedure from the ATS Committee guidelines [8]. All participants were instructed to walk the maximum distance possible in six minutes. Standardized verbal encouragement was not provided, since it has been shown to affect the 6MWT outcomes [24]. The final score was expressed as distance covered in meters (m) during a 6 min period. Height and weight were objectively measured using a portable stadiometer and digital scale with a precision of 0.1 mm and 0.1 kg, respectively. Body mass index was calculated using the following formula: weight (kg)/height (m^2^). Age was self-reported.

### 2.4. Data Analysis

Basic descriptive statistics are presented as the mean and standard deviation (SD). Kolmogorov–Smirnov tests showed that data were normally distributed. Sex differences were examined using the Student’s *t*-test for independent samples. To analyze the correlation between sex, age, BMI and the 6MWT with the VO_2_max, we used Pearson’s coefficient of correlation. For each correlation, we calculated the coefficient (*r*) with a 95% CI and the coefficient of determination (*r*^2^), to examine the % of explained variance in the dependent variable (VO_2_max). A set of multiple regression equations was created to predict the level of VO_2_max. In Model 1, sex (1—men; 2—women) was entered. In Model 2, sex and age (in years) were added. Next to sex and age, BMI (kg/m^2^) was simultaneously entered in Model 3. In Model 4, the final regression predictive equation consisted of sex, age, BMI and the distance covered in the 6MWT (in m). The associations between the dependent (VO_2_max) and the independent variables (sex, age, BMI and the 6MWT) are presented as the unstandardized *β* coefficients with a 95% CI. Based on the model, we created the proposed regression equations to predict the VO_2_max. The statistical analyses were conducted using Statistical Packages for Social Sciences, version 23 (SPSS Inc., Chicago, IL, USA). Two-sided *p*-values were used, and the significance was set at α < 0.05.

## 3. Results

Basic descriptive statistics of the study participants are presented in Table 1. Men were taller, heavier and had higher BMI values compared to women. Also, men exhibited better results in the 6MWT (+3.6%) and the VO_2_max (+11.8%).

Table 2 shows correlations between the 6MWT, demographic (sex and age), anthropometric (BMI) and cardiovascular (RHR) variables with VO_2_max. The strongest correlation between the 6MWT and VO_2_max was observed (*R*^2^ = 50.4%), followed by age (*R*^2^ = 39.7%), BMI (*R*^2^ = 14.4%), and sex (*R*^2^ =9.0%).

A set of regression equations to predict the VO_2_max is shown in Table 3. In Model 1, sex explained 8.4% of the variance in the VO_2_max (*R* = 0.29; *R*^2^ = 0.084; *SEE* = 7.6 mL/kg/min; *p* < 0.001). When age was added to sex in Model 2, the model explained 46.8% of the variance in the VO_2_max (*R* = 0.68; *R*^2^ = 0.468; *SEE* = 5.8 mL/kg/min; *p* < 0.001). When the equation was adjusted for sex, age and BMI in Model 3, the model explained 57.4% of the variance in the VO_2_max (*R* = 0.76; *R*^2^ = 0.574; *SEE* = 5.2 mL/kg/min; *p* < 0.001). In the final model (Model 4), sex, age, BMI and the 6MWT were entered simultaneously, and explained 72.3% of the variance in the VO_2_max (*R* = 0.85; *R*^2^ = 0.723, *SEE* = 3.99 mL/kg/min, *p* < 0.001).

## 4. Discussion

The main purposes of the study were to examine the correlations between the 6MWT and the VO_2_max, and to create predictive equations, taking into account simple demographic (sex and age) and anthropometric (BMI) variables, along with the 6MWT. The findings of this study are that: (i) the 6MWT correlates strongly with the VO_2_max; and (ii) the final predictive regression model with sex, age, BMI and the 6MWT explains 72.3% of the variance in the VO_2_max.

The 6MWT has been widely used as a measure of functional status of an individual to be able to engage in everyday activities [24]. Despite multiple benefits of using the 6MWT in comparison to laboratory testing [24], there has been a surprisingly lack of research examining reference equations using the 6MWT to objectively predict the measured VO_2_max in older adults. Our findings suggest that the 6MWT is strongly correlated with the VO_2_max (*r* = 0.71), and can be hypothetically used to predict the maximal aerobic capacity; however, by adding a few demographic and anthropometric variables, being moderately-to-strongly related with the VO_2_max, the model significantly improves its applicability and validity properties. To date, several predictive regression equations have been developed to estimate VO_2_max in apparently healthy middle-aged and older adults [22,23,24]. A study by Burr et al. [24] showed that when the 6MWT was combined with sex, age, weight and the resting HR, the regression equation was able to account for 72.4% of the variance in the VO_2_max. Similar predictive abilities have been obtained in two other studies, where the inter-correlations between the regression models with the VO_2_max are 76.0% for the total sample [22], 82.0% in men and 79.0% in women, respectively [23]. When the predictive regression equations are based on symptomatic individuals, studies have shown moderate [26] to strong [7,13,21] correlations with an objectively measured VO_2_max. Specifically, a study by Ross et al. [7]. found that the SEE for the fixed and random models ranged between 3.82 and 3.66 mL/kg/min, while the correlation between the 6MWT and the VO_2_max was *r* = 0.59. In a group of patients with chronic heart failure, Adedoyin et al. [26]. found that the distance covered was highly correlated with the VO_2_max (*r* = 0.65), and a mean difference of 0.1 mL/kg/min was observed. Although previous evidence has confirmed moderate-to-strong predictive abilities of regression equations to account for the VO_2_max [7,13,21,22,23,24,25,26], most research has been conducted on a relatively small sample of symptomatic individuals with a great age range, while little evidence has been provided for a specific population of apparently healthy older adults. By using sex, age, BMI and the 6MWT, we were able to develop an accurate estimating equation to predict an objectively measured VO_2_max in older adults aged 60–80 years.

It has been well documented that the use of sub-maximal exercise testing to predict the level of VO_2_max may underestimate the true VO_2_max in patients with cardiovascular diseases [29,30]. The mechanisms related to the variability between the estimated (using the 6MWT) and objectively measured VO_2_max may be explained by the nature of the 6MWT testing. For example, the 6MWT represents a sub-maximal exercise test, where individuals do not often reach their maximal functional capacities, due to possible resting periods in the middle of measurements [8]. Thus, it is possible that the regression equations the 6MWT consisted of would underestimate the actual VO_2_max values [21,26], while other studies overestimated the mean VO_2_max [24]. In our study, the correlation between the estimated and objectively measured VO_2_max was *r* = 0.77 (95% CI 0.73 to 0.82; *p* < 0.001), with a mean difference of 1.35 mL/kg/min (estimated vs. measured VO_2_max: 29.8 ± 6.2 vs. 28.4 ± 7.9 mL/kg/min). Thus, the predictive model developed in our sample of older adults slightly overestimated the mean VO_2_max values. Another factor responsible for the variability in regression equations was the number of independent variables being computed to estimate the VO_2_max. Our intendance was to create an accurate model to predict an objectively measured VO_2_max, based on simple demographic (sex and age), anthropometric (BMI) and distance (the 6MWT) parameters. Most of previous studies have only used the 6MWT [21,22], or the combination of sex [24], age [22,23,24,26], weight [23,24,26], height [23,24], body mass index (BMI) [23] and HR [23,24] to predict the VO_2_max, which was thought to increase the predictive ability of the equations. Finally, the discrepancy between the studies may be due to differences in studied samples, including socio-demographic (the men/women ratio, age, race) and medication management for cardiovascular or respiratory diseases.

The 6MWT has been considered a reliable and valid filed-based method to assess the functional aerobic capacity in both symptomatic [9,10,11,12,13,14,15,16,17,18,19,20,21,26] and asymptomatic [22,23,24] populations. It is a quick, safe and cost-effective tool for measuring a large number of participants in population-based studies, and for intervention purposes [8]. By examining the correlation and predictive ability of the 6MWT, we were able to develop a new equation which accurately estimated the VO_2_max.

This study is not without limitations. First, the design of the study was cross-sectional, and we were unable to determine the causality of the correlation between the 6MWT and the VO_2_max. Second, the sample used in this study did not incorporate a diverse selection of ethnicities (only Caucasian men and women were included). Third, we did not collect any information regarding the physiological and psychological variables, such as a blood sample, HR before and after conducting the 6MWT, or the level of motivation, which might be able to correct the ability of prediction equations to assess the estimated VO_2_max. Fourth, the model was not cross-validated with other independent samples of older adults to determine the sensitivity properties of the prediction model. Finally, the newly developed equation should be tested in weight-loss intervention, where it is speculated that individuals with more favorable body weight can walk at a faster pace.

## 5. Conclusions

In summary, the 6MWT highly correlates with the VO_2_max and, with the combination of sex, age and BMI, the predictive equation is able to account for 72.3% of the variance in an objectively measured VO_2_max. Although we confirm the accuracy of the model, this is one of the first studies conducted among a specific population of apparently healthy older adults.

## Figures and Tables

**Table 1:** Descriptive statistics (mean ± SD) of the study participants (*n* = 233).

Study Variables	Men (*n* = 103)	Women (*n* = 130)	*p* for Sex *
Age (years)	67.8 ± 5.5	66.9 ± 5.7	0.234
Height (cm)	173.8 ± 6.6	162.6 ± 6.4	<0.001
Weight (kg)	82.8 ± 10.5	69.3 ± 12.3	<0.001
BMI (kg/m^2^)	27.0 ± 3.6	26.1 ± 4.0	0.082
The 6MWT (m)	678.3 ± 59.1	653.8 ± 49.9	<0.001
VO_2_max (mL/kg/min)	30.5 ± 9.0	26.9 ± 6.6	<0.001

* *p* < 0.05.

**Table 2:** Correlations between the study variables and the VO_2_max in the study participants (*n* = 233).

Study Variables	*r* Coefficient	95% CI	*p*-Value *
Sex (1—men; 2—women)	−0.30	−0.41 to −0.17	<0.001
Age (years)	−0.63	−0.70 to −0.55	<0.001
BMI (kg/m^2^)	−0.38	−0.49 to −0.27	<0.001
The 6MWT (m)	0.71	0.65 to 0.77	<0.001

* *p* < 0.05.

**Table 3:** Regression equations to predict the VO_2_max in the study participants (*n* = 233).

Study Variables	Unstandardized Estimates (*p*-Value *)	Regression Equation for VO_2_max
**Model 1**		
Constant (intercept)	35.45	35.45 − 4.59 * sex (1—men; 2—women)
Sex (1—men; 2—women)	−4.59 (<0.001)	
**Model 2**		
Constant (intercept)	94.23	94.23 − 4.55 * sex (1—men; 2—women) − 0.87 * age (years)
Sex (1—men; 2—women)	−4.55 (<0.001)	
Age (years)	−0.87 (<0.001)	
**Model 3**		
Constant (intercept)	107.81	107.81 − 5.10 * sex (1—men; 2—women) − 0.80 * age (years) − 0.66 * BMI (kg/m^2^)
Sex (1—men; 2—women)	−5.10 (<0.001)	
Age (years)	−0.80 (<0.001)	
BMI (kg/m^2^)	−0.66 (<0.001)	
**Model 4**		
Constant (intercept)	59.44	59.44 − 3.83 * sex (1—men; 2—women) − 0.56 * age (years) − 0.48 * BMI (kg/m^2^) + 0.04 * the 6MWT (m)
Sex (1—men; 2—women)	−3.83 (<0.001)	
Age (years)	−0.56 (<0.001)	
BMI (kg/m^2^)	−0.48 (<0.001)	
The 6MWT (m)	0.04 (<0.001)	

* *p* < 0.05.

## Data Availability

The raw data used in this study are available from the corresponding author upon a reasonable request.
